# Comparative Study of the Adsorption of Acid Blue 40 on Polyaniline, Magnetic Oxide and Their Composites: Synthesis, Characterization and Application

**DOI:** 10.3390/ma12182854

**Published:** 2019-09-04

**Authors:** Amir Muhammad, Anwar ul Haq Ali Shah, Salma Bilal

**Affiliations:** 1 Institute of Chemical Sciences, University of Peshawar, Peshawar 25120, Pakistan; 2 National Centre of Excellence in Physical Chemistry, University of Peshawar, Peshawar 25120, Pakistan; 3 TU Braunschweig Institute of Energy and Process Systems Engineering, Franz-Liszt-Straße 35, 38106 Braunschweig, Germany

**Keywords:** Acid blue 40 dye, adsorption isotherms, kinetics and thermodynamic study

## Abstract

Conducting polymers (CPs), especially polyaniline (PANI) based hybrid materials have emerged as very interesting materials for the adsorption of heavy metals and dyes from an aqueous environment due to their electrical transport properties, fascinating doping/de-doping chemistry and porous surface texture. Acid Blue 40 (AB40) is one of the common dyes present in the industrial effluents. We have performed a comparative study on the removal of AB40 from water through the application of PANI, magnetic oxide (Fe_3_O_4_) and their composites. Prior to this study, PANI and its composites with magnetic oxide were synthesized through our previously reported chemical oxidative synthesis route. The adsorption of AB40 on the synthesized materials was investigated with UV-Vis spectroscopy and resulting data were analyzed by fitting into Tempkin, Freundlich, Dubinin–Radushkevich (D–R) and Langmuir isotherm models. The Freundlich isotherm model fits more closely to the adsorptions data with R^2^ values of 0.933, 0.971 and 0.941 for Fe_3_O_4_, PANI and composites, respectively. The maximum adsorption capacity of Fe_3_O_4_, PANI and composites was, respectively, 130.5, 264.9 and 216.9 mg g^−1^. Comparatively good adsorption capability of PANI in the present case is attributed to electrostatic interactions and a greater number of H-bonding. Effect of pH of solution, temperature, initial concentration of AB40, contact time, ionic strength and dose of adsorbent were also investigated. Adsorption followed pseudo-second-order kinetics. The activation energy of adsorption of AB40 on Fe_3_O_4_, PANI and composites were 30.12, 22.09 and 26.13 kJmol^−1^ respectively. Enthalpy change, entropy change and Gibbs free energy changes are −6.077, −0.026 and −11.93 kJ mol^−1^ for adsorption of AB40 on Fe_3_O_4_. These values are −8.993, −0.032 and −19.87 kJ mol^−1^ for PANI and −10.62, −0.054 and −19.75 kJ mol^−1^ for adsorption of AB40 on PANI/Fe_3_O_4_ composites. The negative sign of entropy, enthalpy and Gibbs free energy changes indicate spontaneous and exothermic nature of adsorption.

## 1. Introduction

The discovery of conducting polymers in 1977 initiated an interesting field of research. These polymers showcased fascinating physico-chemical properties which made them suitable for numerous applications [1]. Polyaniline, polythiophene, polypyrrole and their derivatives are the most studied conducting polymers [2,3,4,5] and show optical as well as conducting properties due to the presence of π conjugated electrons in their skeleton [6]. Polyaniline (PANI) has gained a lot interest among the conducting polymers family because it can be synthesized easily from low-cost materials. It is highly conductive and possesses good environmental stability [3,7,8].

A number of methods including chemical oxidation, electro-chemical oxidation, enzymatic, interfacial, self-assembling and seeding methods have been applied to synthesize PANI [9,10,11,12,13]. Chemical and electro-chemical oxidation methods are the most common methods which involve the polymerization of aniline in an acidic or basic medium. However, the conducting emeraldine form of PANI is usually synthesized in an acidic environment [14]. PANI has been effectively applied in corrosion protection, batteries, solar cells, supercapacitors and adsorption of heavy metals and dyes from an aqueous solution [3,15,16,17,18]. The suitability of PANI as an adsorbent to remove dyes from an aqueous environment is due to the presence of a large number of amine and imine functional groups which are expected to interact with dyes. The charge transfer induced by doping enables PANI to interact with ionic species through electrostatic interactions [19]. Although PANI has been used widely as an adsorbent for the removal of dyes from water, its performance is restricted due to two main challenges. Firstly, its particles aggregate due to intermolecular interactions, resulting in the decrease of surface area and hence the adsorption capacities [20]. Secondly, acid doped PANI is prone to de-doping due to the evaporation of the small acid molecules at room temperature. This causes a reduction in the surface charge of PANI which ultimately affect the electrostatic interaction between PANI and dye [21].

To overcome these challenges, considerable work has been done in recent years to synthesize composites of PANI with inorganic substances such as Ag, Cd, SiO_2_, TiO_2_, ZnO, MnO_2_ and magnetic oxide (Fe_3_O_4_) [22,23,24,25]. These composites exhibit characteristics electrical, optical, catalytic and mechanical properties that are better than single components in some cases. The composites of PANI and Fe_3_O_4_ have attracted much attention because of easy synthesis and numerous applications in areas such as in biosensors, sensors, solar cells and purification of water [26,27,28,29].

Just like PANI, magnetic oxide also finds applications in drug delivery systems [30], clinical diagnosis [31], efficient hyperthermia for the removal of cancer [32], microwave devices, magnetic resonance imaging (MRI) [33,34] and the removal of heavy metals from an aqueous solution [35,36]. Electric explosion of wire, laser target evaporation and biomineralization are commonly used for controlled size and morphology of Fe_3_O_4_ [37], but the wet chemical methods, like the chemical co-precipitation method [38], sol-gel [39], hydrothermal method [40], gas phase [41], liquid phase [42] and two-phase methods such as microemulsion methods [43] are also used for the preparation of Fe_3_O_4_.

In general, composites of PANI and Fe_3_O_4_ have been synthesized either through in situ formations of magnetic oxide composites in the presence of PANI [44] or polymerization of aniline monomers in the presence of iron oxide. In comparison with the former, the latter strategy gives better results because of the magnetic properties of the resulting hybrid materials [45].

Bhaumik et al. [46] prepared nanofibers composites from metallic nanoparticles and PANI and applied these composites to remove arsenic (V), chromium (VI) and Congo red from an aqueous solution. Different polymer salts (PANI–HNO_3_, PANI–H_2_SO_4_ and PANI–H_3_PO_4_) are reported to use as adsorbents to remove Direct Blue 78 (DB78) from water [47]. The dye uptake was in the order PANI–H_3_PO_4_ > PANI–H_2_SO_4_ > PANI–HNO_3_. The rate of adsorption was decreased as the concentration of DB78 and pH of dye solution increased. The adsorption followed pseudo-second-order kinetics. Cui and co-workers [48], studied the adsorption of Hg (II) onto polyaniline/attapulgite (PANI/ATP) composites. (PANI/ATP) composites were synthesized by the chemical oxidation method. The maximum amount of dye adsorbed was 800 mg/g when the pH of Hg (II) solution was 5.9 and followed pseudo-second-order kinetics.

In the present study, PANI/Fe_3_O_4_ is used as an adsorbent to remove Acid Blue 40 (AB40) from water. The adsorption behaviors of PANI/Fe_3_O_4_ were compared with PANI and Fe_3_O_4_ which were synthesized and tested according to our previous work [49]. The chemical oxidation method was used to synthesize PANI and PANI/Fe_3_O_4_ composites using FeCl_3_·6H_2_O as an oxidant in an acidic medium, while the chemical co-precipitation method was adopted to synthesize Fe_3_O_4_ materials in the basic medium at a temperature of 85–90 °C. All these synthesized materials were characterized through UV-Vis, SEM, FTIR, EDX and surface area measurements. Adsorption study was carried out to determine the effect of pH, initial concentration, temperature, contact time, adsorbent dosage and ionic strength on adsorption phenomenon using UV-Vis spectroscopy. Freundlich, Langmuir, D–R and Tempkin adsorption isotherm models were applied to analyze the adsorption data. The adsorption mechanism was determined on the basis of kinetic study. Thermodynamic aspects of adsorption of AB40 on these materials were also investigated.

## 2. Experiment

### 2.1. Materials

Aniline was purchased from Across and distilled under vacuum. Acid Blue 40 dye, FeCl_3_·6H_2_O and Na_2_SO_4_ (Sigma-Aldrich, St. Louis, MO, USA), Dodecyl benzene sulfonic acid (DBSA) (Across) and FeSO_4_·7H_2_O (Merck, Kenilworth, NJ, USA ) were used without further purification.

### 2.2. Synthesis of PANI

PANI was synthesized via our previously reported chemical oxidation method [49]. Typically, 0.02 M (1.182 mL) aniline was suspended in 50 mL of 0.01 M H_2_SO_4_ solution. To this suspension 0.01 M (0.15 mL) DBSA was added as an emulsifying agent. Then 50 mL of 0.01 M FeCl_3_·H_2_O prepared in 0.01 M H_2_SO_4_ was added drop by drop as an oxidant with constant stirring. After 20 min of continuous stirring, a milky white color suspension turned to light green and then dark green in one hour. The final product was thoroughly washed with acetone and then with double-distilled water until the filtrate became clear. The obtained powder was dried in an oven for 24 h at 60 °C.

### 2.3. Synthesis of Fe_3_O_4_

Fe_3_O_4_ was synthesized by the chemical co-precipitation method by adding 0.15 mL DBSA and 2 M of FeCl_3_·6H_2_O dissolved in 50 mL of 0.1 M NaOH to 0.5 M FeSO_4_·7H_2_O solution. The whole mixture was stirred continuously at 85–90 °C. After 20 min of stirring, 30 mL of 5 M ammonia solution was added at once which turned the color of the reacting mixture to black. The pH of the reacting mixture was kept at 10 during the whole experiment. After two hours of continuous stirring at 85–90 °C, the precipitate was washed with ethanol and double distilled water until the effluent became clear. The black precipitate was dried at 80 °C for 10 h and then annealed at 600 °C for 5 h in a furnace (NEYCRAFT JFF 2000 Fiber Furnace, France).

### 2.4. Synthesis of PANI/Fe_3_O_4_ Composites

PANI/Fe_3_O_4_ composites were synthesized by suspending 0.15 g Fe_3_O_4_ particles in 30 mL of 0.01 M H_2_SO_4_ solution followed by addition of 50 mL (0.02 M) of aniline solution prepared in 0.01 M H_2_SO_4_ and 0.5 mL (0.01) DBSA, respectively. After 30 min of continuous stirring, 50 mL of 0.01 M FeCl_3_·6H_2_O was added as an oxidizing agent. A light green color appeared within the stirring mixture after 20 min of the oxidant’s addition. The color of this mixture turned dark black after about one hour. After continuously stirring for 6 h, the product was separated and washed with acetone and double-distilled water. The clean precipitate was dried in oven at 60 °C for 24 h.

### 2.5. Batch Adsorption Study for Removal of AB40 Dye

Twenty-milliliter solutions of different concentrations between 5–120 mgL^−1^ were prepared from the stock solution of AB40 dye. To these solutions, PANI was added and shacked for about 120 min. These solutions were then filtered to determine the concentration of dye in the filtrate using UV-Visible spectrophotometer and applying Equation (1) [50].

(1) $$q_{e}=\frac{{(C}_{i}{-\;C}_{e})V}{m}$$

where q_e_ (mg g^−1^) refers to adsorption at equilibrium, C_i_ (mg L^−1^) and C_e_ (mg L^−1^) show initial and equilibrium concentration of dye, respectively, m (g) is the mass of adsorbent while V represents the volume of solution in mL. The effect of temperature, contact time, ionic strength, pH and initial concentration of dye solution on the adsorption behavior was studied. The adsorption data were utilized to calculate the thermodynamic and kinetics parameters. The same procedure was employed for studying adsorption of AB40 on Fe_3_O_4_ and PANI/Fe_3_O_4_ composite.

Before the adsorption process, standard solutions of AB40 dye were prepared was in the range 0.005–2 mg L^−1^ and their maximum absorption was determined via UV-Visible spectrophotometer (Perkin Elmer, Waltham, MA, USA). The absorption values were plotted against concentration of the standard dye solutions and calibration curve was obtained according to the Beer–Lambert law. The slope so obtained was used as reference for the determination of concentration in rest of the experiments. The calibration curve is also shown in the supporting files (Figure S1).

After the adsorption of AB40 dye, the adsorbents were put onto the filter paper and washed several times with double-distilled water to run out the adsorbed dye. Then it was washed with 0.1 M NaOH to remove the remaining dye. This process enables the reutilization of the adsorbent.

### 2.6. Characterization

FTIR spectra of the synthesized materials were registered in the spectral range of 400 to 4000 cm^−1^ through a Fourier transmission infrared spectrophotometer (Shimadzu, Tokyo, Japan). X-ray diffraction (XRD) patterns were recorded by using Cu Kα radiations of wavelength 1.5405 Å with the help of a JEOL JDX-3532 (JEOL, Tokyo, Japan). The concentration of dye in the solution and its adsorbed amount onto the synthesized materials were checked through UV-visible spectrophotometer (Perkin Elmer, Buckinghamshire, UK). An energy-dispersive X-ray (EDX) spectrophotometer (Inca 200, Oxford, UK) was utilized to determine the percentage of different elements. Brunauer–Emmett–Teller (BET) surface areas of the composite as well as PANI and Fe_3_O_4_, were determined in the N_2_ atmosphere through adsorption–desorption method with a surface area analyzer model 2200 e Quanta Chrome (Quanta Chrome, Boynton Beach, FL, USA). The surface morphologies were studied through scanning electron microscopy (SEM) (JSM-6490, JEOL, Tokyo, Japan).

## 3. Results and Discussion

### 3.1. Scanning Electron Microscopy (SEM)

**Figure 1:**
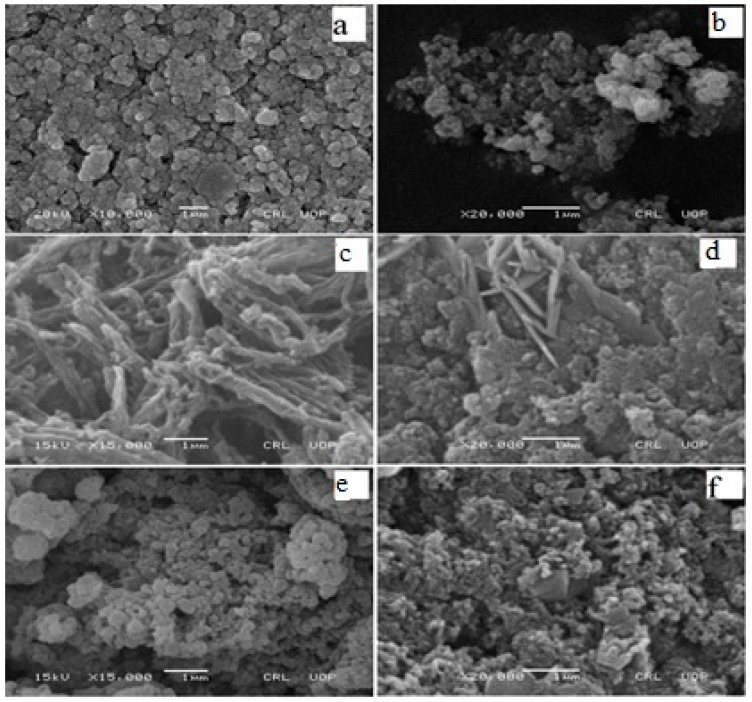
SEM images of magnetic oxide (Fe_3_O_4_), polyaniline (PANI) and PANI/Fe_3_O_4_ composite before (**a**,**c**,**e**) and after (**b**,**d**,**f**) adsorption of acid blue 40 (AB40) dye.

SEM images provide interesting information about surface morphology and size of adsorbent materials under investigation. Figure 1a,b shows SEM images of Fe_3_O_4_ particles before and after adsorption of AB40 dye. The Fe_3_O_4_ particles are round in shape with an average size of 0.15 µm. After adsorption of AB40, the porosity decreases in the agglomerated surface of Fe_3_O_4_ but the average particles size increases to 0.23 µm (Figure 1b). Keyhanian et al. [50] have reported the agglomeration of magnetic particles of Fe_3_O_4_ after adhering of methyl violet dye from an aqueous solution.

Rods- or wires-like porous structure can be seen in the SEM image of PANI with 0.21 µm average diameter of the rods (Figure 1c). These rods are aggregated to each other like fibers. After the adsorption of AB40 (Figure 1d), the morphology of PANI changes to a cauliflower shape with some needle-like structures present on the surface. Such a change in morphology was also reported during the adsorption of anionic dyes on PANI doped with Potash Alum [51]. Figure 1e shows an SEM image of PANI/Fe_3_O_4_ composites. It shows a porous morphology where Fe_3_O_4_ particles have adhered with PANI interconnected rods. Similar morphology was depicted by nanocomposites of PANI/ Fe_3_O_4_ coated on MnFe_2_O_4_ [52]. Just like PANI, morphology of PANI/Fe_3_O_4_ composites also changes after the adsorption of AB40. The dye distributes homogeneously over the surface of composite imparting a broccoli-like appearance to it as shown in Figure 1f.

#### 3.1.1. Optical Studies

**Figure 2:**
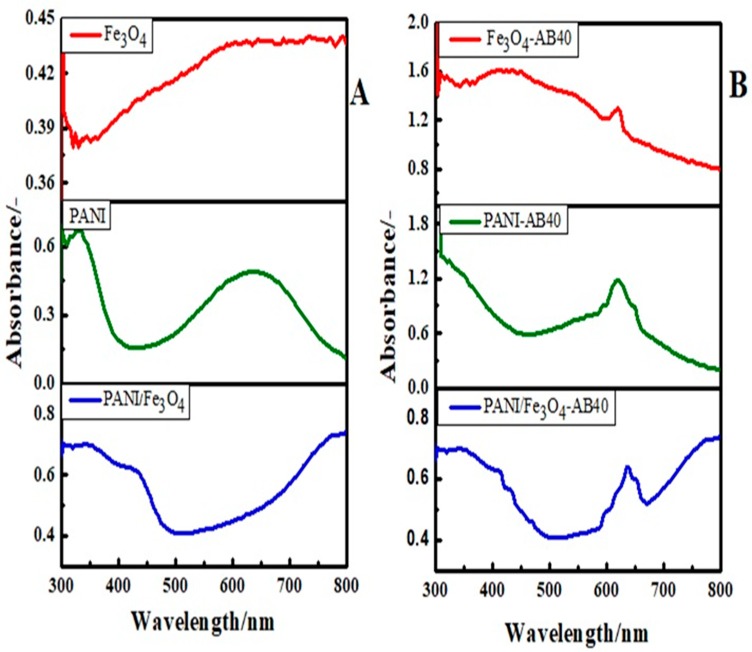
UV-visible spectrum of Fe_3_O_4_, PANI and PANI/ Fe_3_O_4_ composite (**A**) before and (**B**) after adsorption of AB40.

Figure 2A represents the UV-Vis spectra of the synthesized materials before adsorption of the dye. A weak band in the region of 450 nm arises due to the interaction of electromagnetic radiations with the valence electrons of iron in the Fe_3_O_4_. As a result, the valence electron of the metal atom starts to oscillate with the frequency of the electromagnetic source [53]. This phenomenon is known as surface plasmon resonance (SPR). Another band at 485.85 nm is due to the presence of DBSA moiety with Fe_3_O_4_ and closely resembles already reported work [54]. The two characteristic bands of PANI can be observed in its spectrum at 333.91 and 633.42 nm. The band at 633.42 nm is due to charge transfer from the benzenoid ring to the quinoid ring and the band at 333.91 nm is attributed to ᴫ-ᴫ* transitions of the benzenoid ring [55]. In the spectrum of PANI/Fe_3_O_4_ composites, the band at 333.91 nm shows a redshift due to the doping of the benzenoid amine with Fe_3_O_4_ particles. Moreover, the bipolaron band at 633.42 nm is shifted to 773.14 nm suggesting that some physical interactions between PANI and Fe_3_O_4_ particles may exist [56].

Figure 2B represent UV-visible spectra of Fe_3_O_4_, PANI and composite of Fe_3_O_4_ with PANI after adsorption of AB40. One can observe a band in the region of 618–620 nm in all the spectra of Fe_3_O_4_, PANI and composite of Fe_3_O_4_ and PANI which indicates the adsorption of AB40. This band has been demonstrated that AB40 shows strong absorption at 620 nm [57]. The intensity of this band is higher for PANI, which is different from our previous work where more intense peaks, due to adsorption of Basic Blue 3 dye, was observed in the spectrum of PANI/Fe_3_O_4_ composite [49]. The reason can be explained by the fact that in the PANI/Fe_3_O_4_ composite, the positively charged active sites of PANI are covered by Fe_3_O_4_. Moreover, the oxygen of Fe_3_O_4_ behave as negatively charged sites, which may cause repulsion to the negative charge of the anionic dye and hence reduces its adsorption.

#### 3.1.2. Energy Dispersive X-ray (EDX) Study

**Figure 3:**
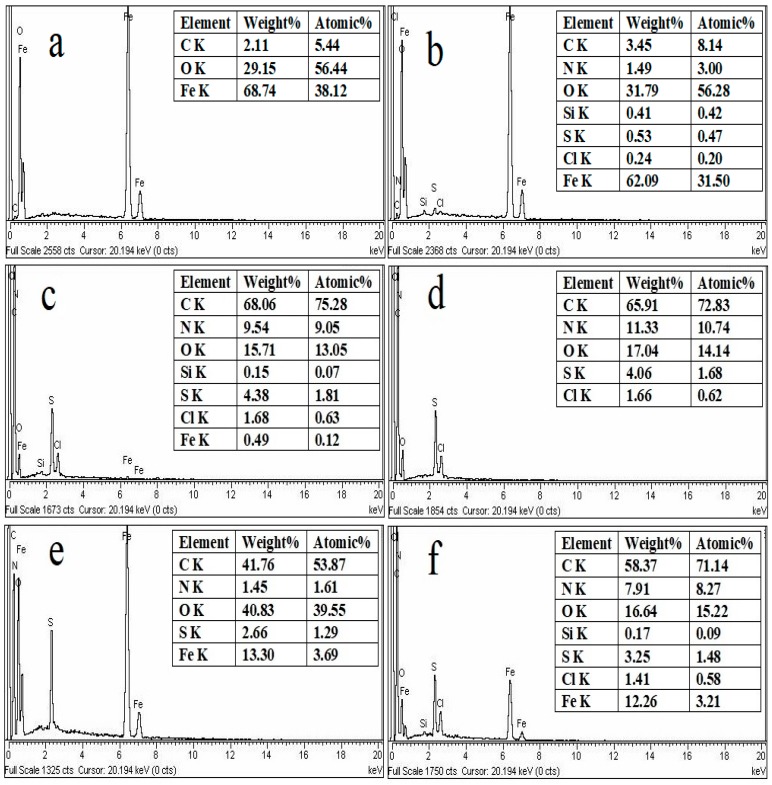
EDX spectra of Fe_3_O_4_, PANI and PANI/Fe_3_O_4_ composites before (**a**,**c**,**e**) and after (**b**,**d**,**f**) adsorption of AB40.

Figure 3 shows the EDX analysis of PANI, Fe_3_O_4_ and PANI/Fe_3_O_4_ composites before and after adsorption of AB40. The weight percent of Fe and O in Fe_3_O_4_ is 68.74 and 29.15, respectively. After the adsorption of AB40, the weight percent of Fe decreases from 68.74 to 62.09, while the percent weight of O and C increases due to the presence of oxygen and carbon in the AB40 texture. Similarly, the appearance of nitrogen and Sulphur in spectrum 3b is more evidence of the adsorption of AB40 onto Fe_3_O_4_, as these elements are present in the dye texture [58]. Figure 3c shows the EDX spectrum of PANI before adsorption of AB40. One can observe a 9.54 percent nitrogen and 68.06 percent carbon by weight in this spectrum. The presence of sulfur and oxygen may be due to the presence of DBSA while Fe and Cl may be due to the presence of FeCl_3_·H_2_O which was used as oxidant. After adsorption of AB40, although weight percent of carbon decreases but weight percent of nitrogen and oxygen increases which shows that AB40 adsorb on PANI (Figure 3d) [59]. In the EDX spectrum of PANI/Fe_3_O_4_ composite, peaks for nitrogen, oxygen, carbon and iron can clearly be observed in Figure 3e, confirming the formation of composites. The sulfur percent by weight is 2.66 and is due to the presence of some moiety of DBSA. After the adsorption of AB40, the weight percent of carbon, nitrogen and sulfur is increased (Figure 3f) [60].

#### 3.1.3. FTIR Study

**Figure 4:**
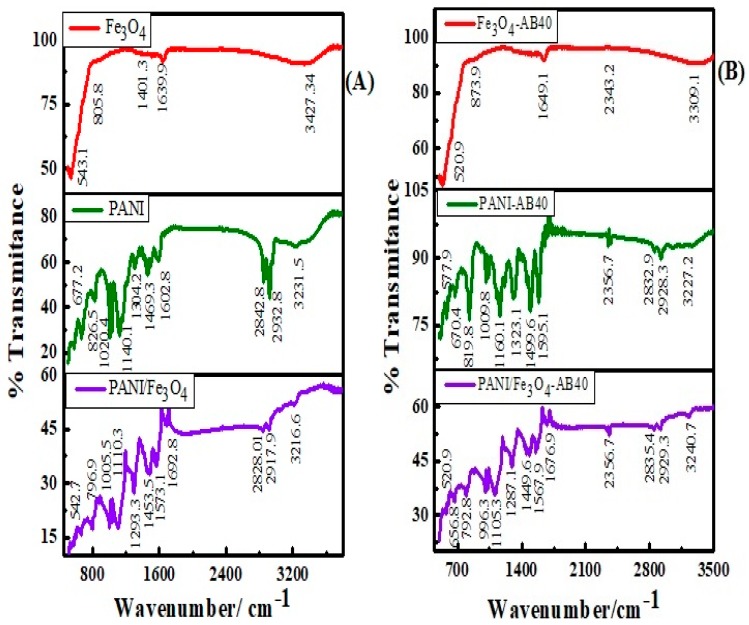
FTIR spectra of Fe_3_O_4_, PANI and PANI/ Fe_3_O_4_ composites before (**A**) and after (**B**) the adsorption of AB40.

Figure 4A,B represent, respectively, FTIR spectra of Fe_3_O_4_, PANI and PANI/Fe_3_O_4_ composite before and after adsorption of AB40. The peak located at 543.1 cm^−1^ is due to the stretching vibration of the Fe–O bond in the Fe_3_O_4_ spectrum [61]. A wide peak at 3427.34 cm^−1^ shows stretching vibrations of –OH group attached to Fe_3_O_4_ surface [62]. The shifting of all peaks towards a lower frequency and the appearance of a very small peak at 2343.2 cm^−1^ in Figure 4B indicates that the AB40 dye comes in contact with Fe_3_O_4_ after adsorption [9,63].

FTIR spectrum of PANI shows –N–H group of secondary amine at 3231.5 cm^−1^. Similarly, the peaks at 2842.8 and 2932.8 cm^−1^ can be attributed to the symmetric and asymmetric stretching vibrations of the C–H bond, respectively. Vivekanandan et al. have reported such asymmetric and symmetric C–H stretching vibrations at 2923.62 and 2825.55 cm^−1^, respectively [9]. Peaks at 1602.8 and 1469.3 cm^−1^ attribute to C=C and C=N stretching vibrations of the benzenoid and quinoid rings. The band at 1304.2 cm^−1^ corresponds to the –C–N^+^ stretching vibrations of the secondary aromatic amine. Similarly, the peaks at 1140.3 and 826.5 cm^−1^ represent the bending vibrations of the aromatic C–H bond in plane and out of plane deformation [64]. The peak at 1020.4 cm^−1^ shows the S=O stretching vibrations of the –SO_3_H group, confirming the presence of DBSA moiety in the PANI texture [65,66]. The peak at 677.2 cm^−1^ shows the out of plane bending vibrations of the C–H bond.

In the spectrum of PANI/Fe_3_O_4_ composites, all peaks are shifted to the low-frequency range in comparison with PANI, indicating a presence of some physical forces between PANI and Fe_3_O_4_ particles. The appearance of the small peak at 542.7 cm^−1^ shows Fe-O stretching, which confirms the formation of PANI/Fe_3_O_4_ composites [67]. After the adsorption of AB40, there is a slight shift of peaks towards a low frequency, both in the spectrum of PANI and PANI/Fe_3_O_4_ composites. Moreover, the appearance of the peak at 2356.7 cm^−1^ shows the adsorption of AB40 dye on PANI and PANI/Fe_3_O_4_ composites [68]. This peak is more intense in the spectrum of AB40 adsorbed on PANI as compared to the PANI/Fe_3_O_4_ composite which is in agreement with the UV-visible study.

#### 3.1.4. Surface Area Study

**Figure 5:**
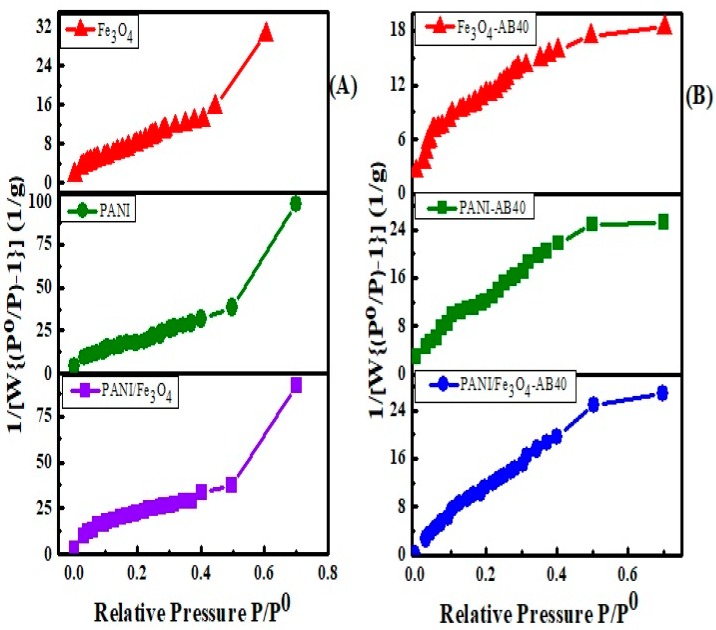
Brunauer–Emmett–Teller (BET) surface area of Fe_3_O_4_, PANI and PANI/Fe_3_O_4_ composites before (**A**) and after (**B**) the adsorption of AB40.

The surface area of adsorbent plays a unique role in the adsorption study. The Brunauer–Emmett–Teller (BET) technique was employed to determine the average pore size radius, pore volume and specific surface area of PANI, Fe_3_O_4_ and PANI/Fe_3_O_4_ composite via nitrogen adsorption–desorption analysis (Figure 5). The results obtained are summarized in Table 1. The data shows that specific surface area of PANI/Fe_3_O_4_ composite is greater than PANI and Fe_3_O_4_ particles [69]. After the adsorption of AB40, the surface area of Fe_3_O_4_, PANI and PANI/Fe_3_O_4_ composite decreases [70]. However, the extent of reduction is more for PANI as compared to Fe_3_O_4_ and PANI/Fe_3_O_4_ composites, showing a greater adsorption of the dye on PANI.

### 3.2. Isotherms Study

**Figure 6:**
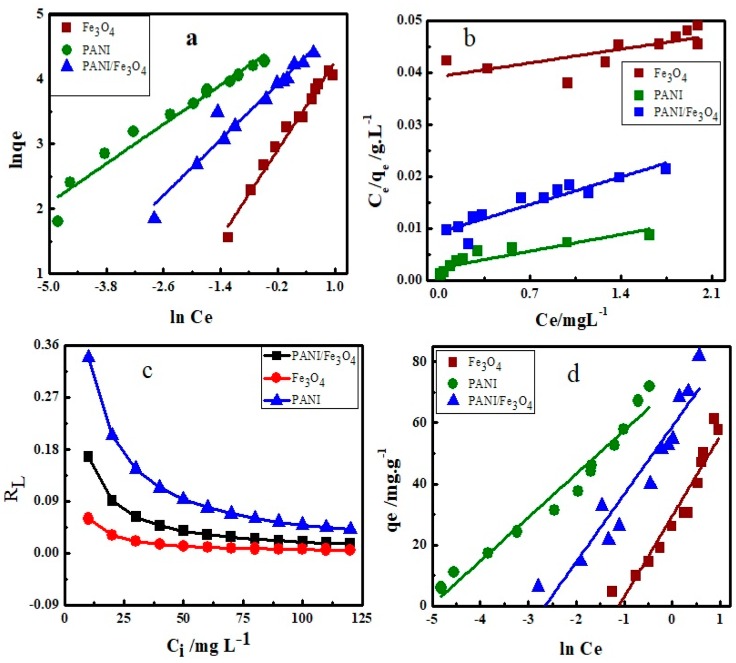

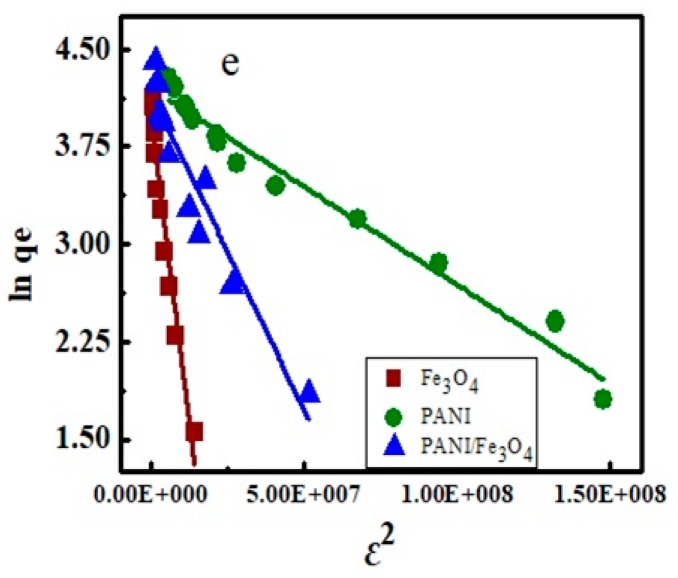
The Isotherm plots (**a**) Freundlich, (**b**) Langmuir, (**c**) Separation factor, (**d**) Tempkin and (**e**) D–R of adsorption of AB40 on Fe_3_O_4_, PANI and PANI/Fe_3_O_4_ composite.

The most important step in the adsorption study is the fitting of adsorption isotherm models to adsorption data in order to describe how interaction occurs between adsorbent and dye. A number of adsorption isotherms are available and have been successfully applied by the earlier researcher to analyze the adsorption data [71]. In this study, four adsorption isotherms models, namely Freundlich, Tempkin, Langmuir and Dubinin–Radushkevich (D–R) were tested. Adsorption parameters so calculated have been summarized in Table 2. The correlation factor *R*^2^, indicates that Freundlich adsorption isotherm equation fit more closely to the adsorption data. The linearized form of Freundlich adsorption equation is expressed in Equation (2);

(2) $${\mathrm{lnq}}_{e}{=\mathrm{lnk}}_{f}+\frac{1}{n}{\mathrm{lnc}}_{e}$$

where q_e_ (mg g^−1^) and C_e_ (mg L^−1^) are the solid and liquid phase equilibrium concentration of dye. K_f_ is constant, and is known as the Freundlich constant and 1/n is the slope obtained by plotting lnq_e_ vs. C_e_ shown in Figure 6A. The values of 1/n vary due to heterogeneity of the adsorbing materials. The values of 1/n shows favorable (0<1/n<1), unfavorable (1/n > 1) or irreversible (1/n = 0) adsorption. However, if its value is unity, the system is at equilibrium and will show heterogeneity [72]. In the present work, the values of 1/n calculated from Freundlich for Fe_3_O_4_, PANI and PANI/Fe_3_O_4_ composites are 0.126, 0.504 and 0.723 respectively showing favorable physical adsorption [73].

The data were also fitted in the Langmuir adsorption isotherm equation (Equation (3)) as shown below;

(3) $$\frac{c_{e}}{q_{e}}=\frac{1}{q_{\max}k_{L}}+\frac{1}{q_{\max}}c_{e}$$

where C_e_ (mg L ^−1^) and q_e_ (mg g^−1^) indicates the concentration of dye and amount of dye adsorbed per gram of adsorbent at equilibrium, respectively. Similarly, K_L_ (mg L^−1^) represent the Langmuir constant related to adsorption energy and q_max_ (mg g^−1^) is the maximum adsorption capacity of adsorbing materials which can be calculated from the slope. The maximum adsorption capacity of AB40 onto Fe_3_O_4_, PANI and PANI/Fe_3_O_4_ composites are 130.5, 264.9 and 216.9 mg g^−1^, respectively, as compared in Table 3A. The dimensionless constant (R_L_) also called separation factor, expresses essential features of the Langmuir isotherm and is represented by Equation (3a).

(3a) $$R_{L}=\frac{1}{{(1+K}_{L}C_{i})}$$

where C_i_ (mg L^−1^) is the initial concentration of AB40. Values of R_L_ indicate that isotherm is either favorable (1 > R_L_ > 0), linear (R_L_ = 1), irreversible (R_L_ = 0) or unfavorable (1 < R_L_) [74]. In the present study, the values of R_L_ range from 0.00525 to 0.34988 as depicted in Figure 6c, which shows that adsorption of AB40 onto Fe_3_O_4_, PANI and PANI/Fe_3_O_4_ composites is favorable at low concentration [75].

The Tempkin isotherm is also an important isotherm model and has been used by researchers to analyze their adsorption data [76,77]. The Tempkin isotherm assumes that due to interactions of the dye with the adsorbent, the adsorption decreases linearly and is characterized by binding energies. It is represented by the following equation (Equation (4));

(4) $$q_{e}{=\beta \mathrm{lnk}}_{T}{+\beta \mathrm{lnc}}_{e}$$

where C_e_ (mg L^−1^), q_e_ (mg g^−1^) and K_T_ (L g^−1^) are equilibrium concentration, equilibrium adsorption and binding constant at equilibrium. It is obtained by plotting q_e_ vs. lnC_e_ (Figure 6d). The constant β, considers the interaction between adsorbent and dye (Equation (4a)).

(4a) $$\beta \;=\frac{\mathrm{RT}}{b}$$

where b is the Tempkin isotherm constant of binding energy (J mol^−1^K^−1^). The correlation factors (*R*^2^) given in Table 2 show that the Tempkin isotherm also fit the adsorption data. The values of K_T_ show that there is strong interaction between AB40 and PANI as compared to Fe_3_O_4_ and PANI/Fe_3_O_4_ composites (Table 2).

Dubinin–Radushkevich (D–R) as expressed in Equation (5) was also fitted to the adsorption data.

(5) $${\mathrm{lnq}}_{e}{=\mathrm{lnq}}_{s}-\beta \varepsilon^{2}$$

where q_e_ is the amount of dye in mg adsorbed per gram of adsorbent (mg g^−1^), β (mol^2^ K^−1^J^−2^) is the activity coefficient useful in obtaining the mean adsorption energy E_ad_ (kJ mol^−1^), q_s_ is the adsorption maximum, and ε is Polanyi potential. ε and E_ad_ are expressed by Equations (5a) and (5b) respectively.

(5a) $$\varepsilon \;=\;\mathrm{RTln}(1+\frac{1}{c_{e}})$$

(5b) $$E_{\mathrm{ad}}=\frac{1}{\sqrt{1\;-\;2\beta }}$$

where R is the gas constant which has a value of 8.314 (J mol^−1^ K^−1^) and T is the kelvin temperature.

D–R adsorption model is a unique model used to differentiate between the chemical and physical adsorption on the basis of adsorption energy. In early research, it has been demonstrated that if the value of adsorption energy is less than 40 kJ mol^−1^, the adsorption is physical [78]. In the present work, the values of E_ad_, calculated by the Equation (5b) are less than 40 kJ mol^−1^ for adsorption of AB40 and PANI as compared to Fe_3_O_4_ and PANI/Fe_3_O_4_ composites showing physical adsorption as shown in Table 2.

### 3.3. Effect of Contact Time and Temperature on Adsorption

**Figure 7:**
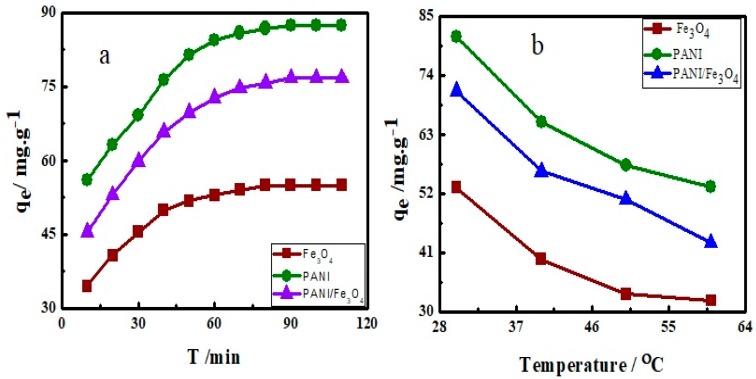
Effect of (**a**) time and (**b**) temperature on adsorption of AB40 onto Fe_3_O_4_, PANI and PANI/Fe_3_O_4_ composite.

The contact time between adsorbent and dye is of great interest in the adsorption process. The optimum time of equilibrium was determined by adding 0.0340 ± 0.0001 g of each Fe_3_O_4_, PANI and PANI/Fe_3_O_4_ composite to 20 mL of AB40 (50 mg L^−1^) in a series of experiments and was shacked at 150 rpm at 30 °C. The adsorption data so obtained was plotted as a function of time (Figure 7a). The graph shows that adsorption is very fast in the initial 10–15 min. The initial fast adsorption is due to a strong interaction between active sites of adsorbents and dye molecules. After 40–50 min, adsorption rate of dye become constant due to filling of active sites on the surface of adsorbents. This time period is defined as the dynamic equilibrium time. At the equilibrium time, rate of adsorption and desorption occurs simultaneously with the same speed [79]. Maximum adsorption of AB40 on Fe_3_O_4_, PANI and PANI/Fe_3_O_4_ composite is observed at 30 °C indicating exothermic nature (Figure 7b).

### 3.4. Effect of pH on Adsorption

**Figure 8:**
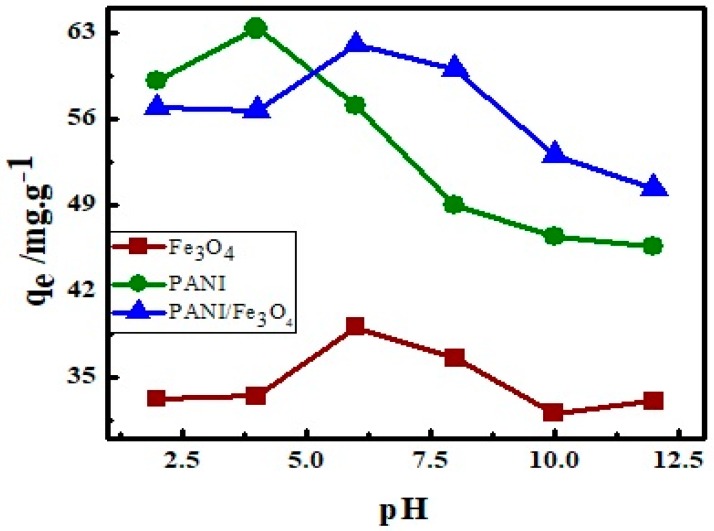
The effect of solution pH on adsorption of AB40 on PANI, Fe_3_O_4_ and PANI/Fe_3_O_4_ composite.

The pH of the dye solution plays a unique role in adsorption process. In the present work, the effect of pH on adsorption was investigated between 2–12. Results so obtained are plotted as adsorption versus pH (Figure 8). The plot indicates that adsorption of AB40 is high in acidic medium on all three adsorbents. When at a low pH, the backbone of adsorbents is positively charged and the active sites like Fe–O and –C=N are protonated. These positively charged sites have a strong interaction with the negatively charged sites of AB40 dye and hence enhance the adsorption. On the other hand, in a basic medium, the deprotonation of Fe–O–H and –C–N–H will create a negative charge in these groups which will repel the negatively charged sites of dye electrostatically, thus adsorption reduces [80].

### 3.5. Effect of Ionic Strength on Adsorption

**Figure 9:**
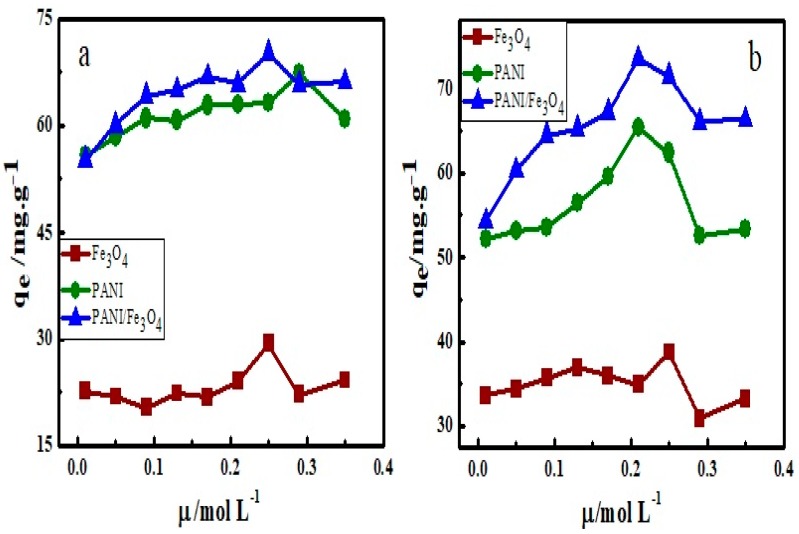
The effect of ionic strength of (**a**) Na_2_SO_4_.7H_2_O and (**b**) CaCl_2_.2H_2_O on adsorption of AB40 onto Fe_3_O_4_, PANI and PANI/Fe_3_O_4_ composite.

The effluent of industrial water also contains several ions. Therefore, the presence of these ions will also affect the adsorption process. In the present study, ionic strength effect of sodium sulfate and calcium chloride on adsorption has been studied in the pH between 5–6. The adsorption data so obtained are plotted against ionic strength (Figure 9). The plots (Figure 9a,b) show that adsorption of AB40 on PANI, Fe_3_O_4_ and PANI/Fe_3_O_4_ composite increases with an increase in ionic strength. This can be attributed to the fact that when both dye and adsorbent have similar charges, an increase in ionic strength will increase adsorption. This effect is more prominent in the adsorption of AB40 on PANI/Fe_3_O_4_ composite as compared to pristine PANI, because PANI/Fe_3_O_4_ composite contains a greater number of sites with a lone pair of electrons which behave as negatively charged groups [81]. Moreover, the significant increase in the adsorption of AB40 by increasing the ionic strength can be attributed to the dimerization of dye. A number of intermolecular forces like dipole-dipole, ion-dipole and Van der Waals forces have been suggested as the cause of the dimerization. Alberghina and co-workers have observed such type of dimerization while studying salts and temperature effect on adsorption of reactive dyes onto activated carbon [51].

### 3.6. Effect of Adsorbent Dosage on Adsorption

**Figure 10:**
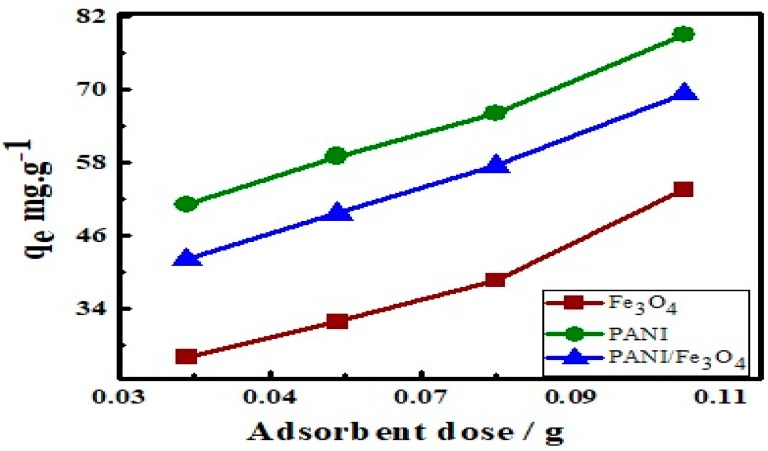
Effect of adsorbent dosage on adsorption of AB40 onto Fe_3_O_4_, PANI and PANI/Fe_3_O_4_ composite.

To investigate the effect of adsorbent dosage on adsorption, 0.034, 0.045, 0.075 and 0.1 g of each Fe_3_O_4_, PANI and PANI/Fe_3_O_4_ composite were added to 100 mg L^−1^ of AB40 separately and shook at 150 rpm at 30 °C and the amount adsorbed was noted (Figure 10). An increase in the adsorption of dye was observed by increasing the adsorbent dose. Initially the rate of adsorption is fast due to greater number of active site and splitting effect of the flux between adsorbents and dye [82].

### 3.7. Adsorption Kinetics

Four kinetic equations namely pseudo 1st order, pseudo 2nd order, Elovich model and intra-particle diffusion models were used to analyze the adsorption data. The relationship between the amount of dye adsorbed on adsorbents and adsorption time was determined. Pseudo-first-order and pseudo-second-order equations are expressed in Equations (6) and (7) respectively as below;

(6) $${\ln(q}_{e}{-\;q}_{t}{)\;=\;\mathrm{lnq}}_{e}{-\;k}_{1}t$$

(7) $$\frac{t}{q_{t}}=\frac{1}{k_{2}q_{e}{}^{2}}+\frac{t}{q_{e}}$$

where q_e_ (mg g^−1^) is the equilibrium adsorption and q_t_ (mg g^−1^) is the amount of dye adsorbed after time t (min). K_1_ (min^−1^) and K_2_ (g mg^−1^ min^−1^) are the rate constants of pseudo-first-order and pseudo-second-order equations respectively.

The Elovich kinetic model can be expressed as shown below in Equation (8);

(8) $$q_{t}=\;\frac{1}{\beta }\ln(\alpha \beta )\;+\;\frac{1}{\beta }\mathrm{lnt}$$

where α (mg g^−1^ min^−1^) shows an initial rate of adsorption and β (mg g^−1^) is the desorption constant relating to the activation energy and the extent of surface coverage.

Intra-particle diffusion model is expressed in Equation (9);

(9) qt= kdt12+ c

where K_d_ (g mg^−1^ min^−1/2^) represent the rate of diffusion constant and C (mg g^−1^) is the constant of boundary layer thickness.

**Figure 11:**
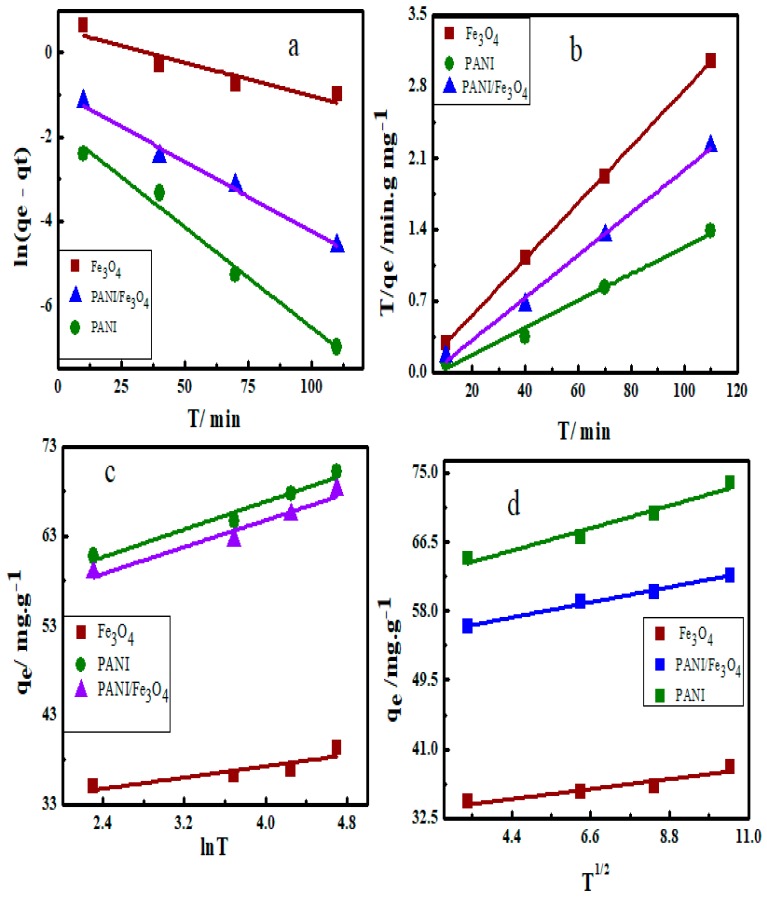
The Kinetics model (**a**) pseudo-first-order, (**b**) pseudo-second-order, (**c**) Elovich model and (**d**) intra-particle diffusion model for adsorption of AB40 on Fe_3_O_4_, PANI and PANI/Fe_3_O_4_ composite.

The fitted curves of adsorption of AB40 onto Fe_3_O_4_, PANI and PANI/Fe_3_O_4_ composites are shown in Figure 11. The parameters of kinetics are summarized in Table 3 and Table 4. The correlation factor of pseudo-first-order (*R*^2^), are 0.812, 0.885 and 0.881 for adsorption of AB40 onto Fe_3_O_4_, PANI and PANI/Fe_3_O_4_ composites. These values indicate that adsorption of AB40 does not follow pseudo-first-order kinetics [83]. Similarly *R*^2^ of Elovich model for PANI/Fe_3_O_4_ composites is 0.707 and intra-particle diffusion model for Fe_3_O_4_ is 0.864 indicating that these models also do not fit well for the adsorption data of AB40 on all of the three adsorbents [84]. *R*^2^ values of pseudo-second-order equation show that the adsorption kinetics are more accurately described by this model (Table 3). Moreover, the q_e_ values calculated by the pseudo-second-order equation agree more closely with the adsorption isotherm values [85].

### 3.8. Adsorption Mechanism

Two routes can be proposed for the adsorption of AB40 on the surface of PANI salt and PANI/Fe_3_O_4_ composite. In the first one, electrostatic interaction may occur between the molecules of AB40 and PANI. The second one involves the formation of an H-bond between the dye and –NH group of PANI. H-bond formation is also possible between AB40 and –OH group present on the surface of Fe_3_O_4_.

The electrostatic interactions are based on the fact that when dye is dissolved in water it splits into positively and negatively charged ions (Dye-SO_3_^−^). These negatively charged anions (Dye-SO_3_^−^) interact with positively charged sites (–^+^NH–) on PANI surface. The enhancement of dye adsorption in acidic medium is good evidence of electrostatic interaction expressed in Section 3.4. Existence of physical forces (H-bond) is also supported by FTIR spectra shown in Figure 4B. After adsorption of AB40, all peaks in the spectra of PANI and PANI/Fe_3_O_4_ composite are shifted towards low-frequency values. Moreover, appearance of peak at 2356.7 cm^−1^ shows existence of AB40 adheres to the surface of PANI and PANI/Fe_3_O_4_ composites [72].

### 3.9. Thermodynamics of Adsorption

**Figure 12:**
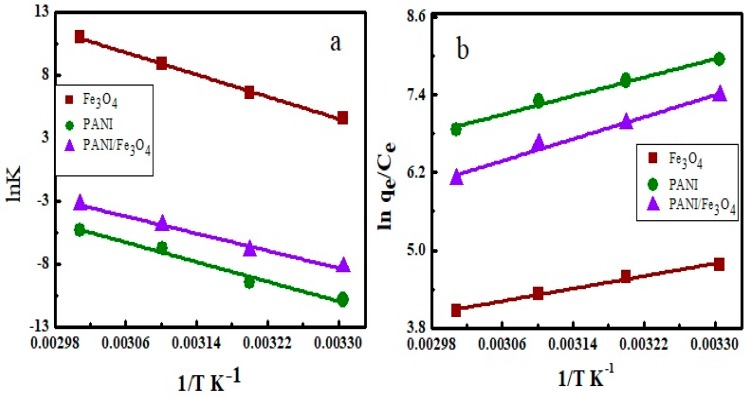
(**a**) Arrhenius plot and (**b**) van’t Hoff plot for calculation of activation energy and thermodynamic parameters.

The nature of adsorption can be described well with thermodynamic parameters like Gibbs free energy, change in enthalpy and change in entropy. Values of Gibbs free energy were calculated by the equation shown below (Equation (10));

(10) $${\Delta G}^{}{\;=\;-\mathrm{RTlnK}}_{e}$$

where K_e_ is the equilibrium constant, R is the gas constant having the value of 8.314 J K^−1^ mol^−1^ and T represents the Kelvin temperature. The negative sign of ∆G values shows that the adsorption of AB40 onto Fe_3_O_4_, PANI and PANI/ Fe_3_O_4_ composites are spontaneous (Table 5). The values of ∆G which range from −20 to zero kJ mol^−1^ show physical adsorption [47]. The values of ∆H and ∆S were calculated from the slope and intercept of van’t Hoff equation respectively by plotting lnk_e_ vs. 1/T (Figure 12b). The van’t Hoff equation is expressed as below;

(11) $${\mathrm{lnK}}_{e}=-\frac{{\Delta H}^{}}{\mathrm{RT}}+\frac{{\Delta S}^{}}{R}$$

(11a) $$K_{e}=\;\frac{q_{e}}{c_{e}}$$

where q_e_ (mg g^−1^) is the adsorption maximum and C_e_ (mg L^−1^) is the concentration of dye at equilibrium. The negative values of ∆H and ∆S shown in Table 4 show that adsorption is exothermic and correlate to the effect of temperature on adsorption expressed in Section 3.3. Activation energy also expresses the nature of adsorption. Its values are calculated from the slope of Arrhenius equation by plotting lnK vs. 1/T shown in Figure 12b. The Arrhenius equation is expressed as below;

(12) $$\mathrm{lnk}\;=\;\mathrm{lnA}\;-\;\frac{\mathrm{Ea}}{\mathrm{RT}}$$

where K is the rate constant, A is Arrhenius constant, Ea is the activation energy, R is the general gas constant and T is kelvin temperature. The activation energy of adsorption of AB40 onto Fe_3_O_4_, PANI and PANI/Fe_3_O_4_ composites are 30.12, 22.09 and 26.13 kJ mol^−1^ showing physical adsorption. Ozcan and co-workers have demonstrated that physical adsorption is characterized by the activation energy values range from 5 to 40 kJ mol^−1^ and its higher values (40–800) kJ mol^−1^ express chemical adsorption [85].

## 4. Conclusions

PANI, Fe_3_O_4_ and their composite can effectively be utilized for the removal of AB40 dye from aqueous environment. The comparison of adsorption behavior of the three materials for the uptake of AB40 reveals that the dye interaction with PANI was higher than both Fe_3_O_4_ and composites materials. This enhancement in adsorption on PANI can be attributed to the electrostatic interaction between oppositely charged sites of PANI and AB40. Greater number of active sites leading to physical forces also enhanced the adsorption of dye on PANI. In case of PANI/Fe_3_O_4_ composites the lone pair electrons present on the oxygen causes repulsive interaction with the negatively charged dye and reduces the adsorption. This fact was confirmed in the effect of ionic strength on adsorption where PANI/Fe_3_O_4_ composites showed higher adsorption than pristine PANI. The maximum amount of dye adsorbed on PANI, Fe_3_O_4_ and PANI/Fe_3_O_4_ composites were 264.9, 130.5 and 216.9 mg g^−1^, respectively. The enhancement of adsorption on PANI was also supported by its smaller value of activation energy than Fe_3_O_4_ and PANI/Fe_3_O_4_ composites. Freundlich adsorption isotherm model fitted more closely with the adsorption data. The adsorption was high in acidic conditions and followed pseudo-second-order kinetics. The negative sign of the values of enthalpy changes, entropy changes and Gibbs free energy changes confirmed spontaneous and exothermic nature of adsorption.

## Figures and Tables

**Table 1:** Surface area, average pore volume and pore radius of PANI, Fe_3_O_4_ and PANI/Fe_3_O_4_ composites.

Status	Materials	Surface Area (m^2^/g)	BJH Average Pore Radius (A^0^)	BJH Pore Volume (cc/g)
Before adsorption	Fe_3_O_4_	71.314	15.749	0.053
	PANI	95.423	16.565	0.049
	PANI/Fe_3_O_4_	98.184	15.501	0.069
After adsorption	Fe_3_O_4_-AB40	53.707	13.334	0.033
	PANI-AB40	43.938	11.743	0.043
	PANI/Fe_3_O_4_-AB40	65.269	12.804	0.036

**Table 2:** Summary of parameters calculated from adsorption isotherms models.

Adsorbents	Adsorption Isotherms												
	Freundlich			Langmuir				Tempkin			D-R		
	1/n	Kf	R^2^	q_max_	K_L_	R_L_	R^2^	ß_T_	K_T_	R^2^	q_S_	E_ads_	R^2^
Fe_3_O_4_	0.126	22.88	0.933	130.5	0.195	0.059	0.499	26.38	3.050	0.917	98.83	3.199	0.933
PANI	0.504	98.21	0.971	264.9	1.579	0.339	0.773	14.24	153.6	0.957	166.7	23.63	0.971
PANI/Fe_3_O_4_	0.723	58.99	0.946	216.9	0.499	0.167	0.859	22.15	14.17	0.902	134.5	11.29	0.909

**Table 3:** Kinetics parameters for adsorption of AB40 on Fe_3_O_4_, PANI and PANI/Fe_3_O_4_ composite based on pseudo-first-order and pseudo-second-order equations.

	Pseudo 1st Order			Pseudo 2nd Order		
Adsorbents	K_1_ (min^−1^)	qe (mg g^−1^)	R^2^	K_2_ (g mg^−1^ min^−1^)	qe (mg g^−1^)	R^2^
Fe_3_O_4_	-0.015	1.765	0.812	0.0665	126.3	0.999
PANI	-0.033	4.823	0.885	0.0028	258.8	0.983
PANI/ Fe_3_O_4_	-0.047	2.495	0.881	0.0213	207.3	0.994

**Table 4:** Kinetics parameters for adsorption of AB40 on Fe_3_O_4_, PANI and PANI/Fe_3_O_4_ composite with Elovich model and intra-particle diffusion model.

	Elovich Model			Intra Particle Diffusion Model		
Adsorbents	α (mg g^−1^min^−1^)	β (g mg^−1^)	R^2^	k_d_ (g mg^−1^min^−1/2^)	C (mg g^−1^)	R^2^
Fe_3_O_4_	131.9	0.258	0.911	0.556	32.45	0.864
PANI	439.8	0.637	0.929	1.257	61.89	0.916
PANI/ Fe_3_O_4_	378.7	0.267	0.707	0.847	53.47	0.917

**Table 5:** Activation energy and thermodynamic parameters of AB40 adsorption.

Adsorbents	∆H (kJ mol^−1^)	∆S (kJ mol^−1^)	∆G (kJ mol^−1^)	Ea (kJ mol^−1^)
Fe_3_O_4_	−6.077	−0.026	−11.93	30.12
PANI	−8.993	−0.032	−19.87	22.09
PANI/Fe_3_O_4_	−10.62	−0.054	−19.75	26.13

## Supplementary Materials

The following are available online at https://www.mdpi.com/1996-1944/12/18/2854/s1, Figure S1: UV-Visible calibration curve of AB40.
