# Supplementary figures and images for: Comparative Study of the Adsorption of Acid Blue 40 on Polyaniline, Magnetic Oxide and Their Composites: Synthesis, Characterization and Application
Source: Materials (Basel). 2019 Sep 4;12(18):2854. doi: 10.3390/ma12182854 (PMC6765991; doi:10.3390/ma12182854)

## Supplementary Materials

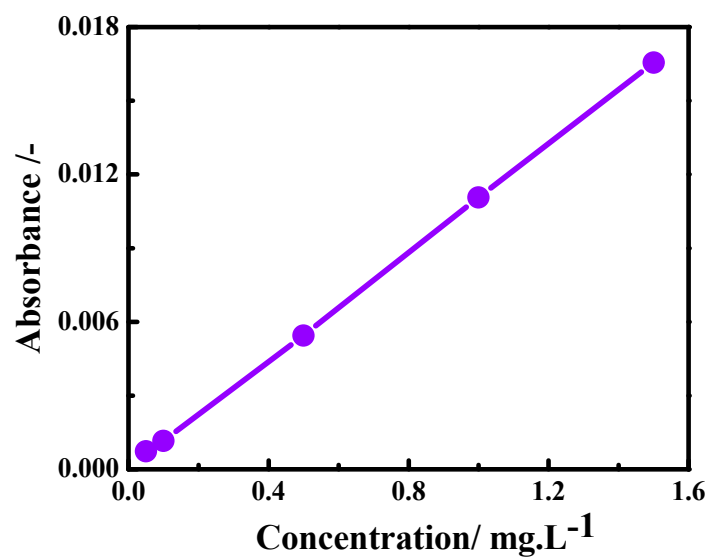

**Figure S1.** UV-Visible calibration curve of AB40.

Supplement: Supplementary file 1 [file materials-12-02854-s001.pdf]
